# Effects of exogenous lactate on lipid, protein, and glucose metabolism—a randomized crossover trial in healthy males

**DOI:** 10.1152/ajpendo.00301.2023

**Published:** 2024-02-07

**Authors:** Mette G. B. Pedersen, Nikolaj Rittig, Maj Bangshaab, Kristoffer Berg-Hansen, Nigopan Gopalasingam, Lars C. Gormsen, Esben Søndergaard, Niels Møller

**Affiliations:** ^1^Steno Diabetes Center Aarhus, Aarhus University Hospital, Aarhus, Denmark; ^2^Medical Research Laboratory, Aarhus University, Aarhus, Denmark; ^3^Department of Cardiology, Aarhus University Hospital, Aarhus, Denmark; ^4^Department of Nuclear Medicine & PET Centre, Aarhus University Hospital, Aarhus, Denmark

**Keywords:** energy expenditure, insulin sensitivity, lactate, lipolysis, protein metabolism

## Abstract

Lactate may inhibit lipolysis and thus enhance insulin sensitivity, but there is a lack of metabolic human studies. This study aimed to determine how hyperlactatemia affects lipolysis, glucose- and protein metabolism, and insulin sensitivity in healthy men. In a single-blind, randomized, crossover design, eight healthy men were studied after an overnight fast on two occasions: *1*) during a sodium-lactate infusion (LAC) and *2*) during a sodium-matched NaCl infusion (CTR). Both days consisted of a 3-h postabsorptive period followed by a 3-h hyperinsulinemic-euglycemic clamp (HEC). Lipolysis rate, endogenous glucose production (EGP), and delta glucose rate of disappearance (ΔRd_glu_) were evaluated using [9,10-^3^H]palmitate and [3-^3^H]glucose tracers. In addition, whole body- and forearm protein metabolism was assessed using [^15^N]phenylalanine, [^2^H_4_]tyrosine, [^15^N]tyrosine, and [^13^C]urea tracers. In the postabsorptive period, plasma lactate increased to 2.7 ± 0.5 mmol/L during LAC vs. 0.6 ± 0.3 mmol/L during CTR (*P* < 0.001). In the postabsorptive period, palmitate flux was 30% lower during LAC compared with CTR (84 ± 32 µmol/min vs. 120 ± 35 µmol/min, *P* = 0.003). During the HEC, palmitate flux was suppressed similarly during both interventions (*P* = 0.7). EGP, ΔRd_glu_, and *M* value were similar during LAC and CTR. During HEC, LAC increased whole body phenylalanine flux (*P* = 0.02) and protein synthesis (*P* = 0.03) compared with CTR; LAC did not affect forearm protein metabolism compared with CTR. Lactate infusion inhibited lipolysis by 30% under postabsorptive conditions but did not affect glucose metabolism or improve insulin sensitivity. In addition, whole body phenylalanine flux was increased. Clinical trial registrations: NCT04710875.

**NEW & NOTEWORTHY** Lactate is a decisive intermediary metabolite, serving as an energy substrate and a signaling molecule. The present study examines the effects of lactate on substrate metabolism and insulin sensitivity in healthy males. Hyperlactatemia reduces lipolysis by 30% without affecting insulin sensitivity and glucose metabolism. In addition, hyperlactatemia increases whole body amino acid turnover rate.

## Graphical abstract

**Figure:**
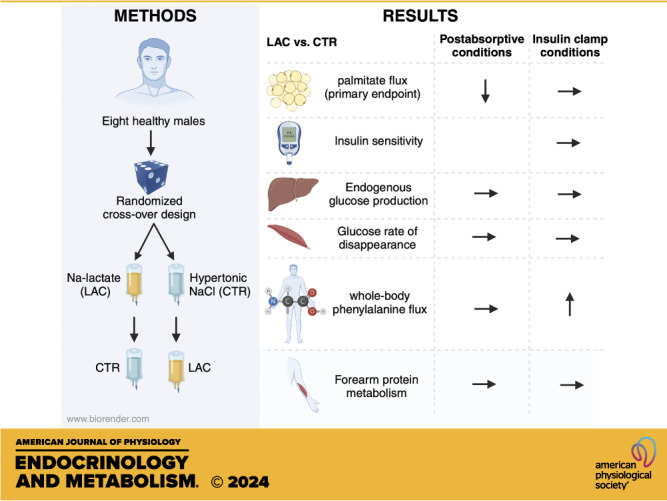

## INTRODUCTION

Lactate is the end product of glycolysis and an essential metabolite in intermediary metabolism. It is produced in tissues with glycolytic capacity and serves as an important energy source through its oxidation to pyruvate and as a key gluconeogenic precursor. Moreover, lactate is a signaling molecule and the primary ligand of the G-protein-coupled receptor 81 (GPR81/HCA1), which is highly expressed in human adipose tissue (1, 2).

Exercise augments energy demand. Initially, this requirement is met by increased free fatty acid (FFA) oxidation (3). As exercise intensity increases, carbohydrate metabolism becomes the primary energy source (4). The shift from FFA to glucose metabolism during high-intensity exercise is associated with a marked rise in plasma lactate concentrations (5). Therefore, it has been suggested that lactate may exert a direct inhibitory effect on lipolysis. It is well-established that lactate has antilipolytic properties in cell- and animal models (2, 6–9). However, the results from clinical trials have been inconsistent and generally without using lipid tracers (10–13). In addition, studies outside the context of exercise are scarce. In an exercise study with healthy volunteers, lactate infusion hampered the exercise-related increase in free fatty acid and glycerol (10). Yet, in another study, local lactate administration in abdominal subcutaneous adipose tissue during exercise in healthy men failed to inhibit the exercise-related increase in FFA concentrations (12). Furthermore, elevated lactate concentrations did not affect free fatty acid- or glycerol concentrations in healthy volunteers at rest (11).

The current study aimed to investigate whether isolated hyperlactatemia within a physiological range inhibits whole body lipolysis measured by a palmitate radiotracer in healthy men.

We hypothesized that lipolysis would be lower during lactate administration, enhancing insulin sensitivity. In addition, we aimed to determine the effect of isolated hyperlactatemia on glucose- and protein metabolism.

## METHODS

### Ethics

The study was approved by the Central Regional Ethical Committee of Jutland, Denmark (1-10-72-180-20), registered at the Danish Data Protection Agency, and on Clinicaltrial.gov (NCT04710875). Written informed consent was obtained from each volunteer before inclusion.

### Volunteers

**Figure A1.**
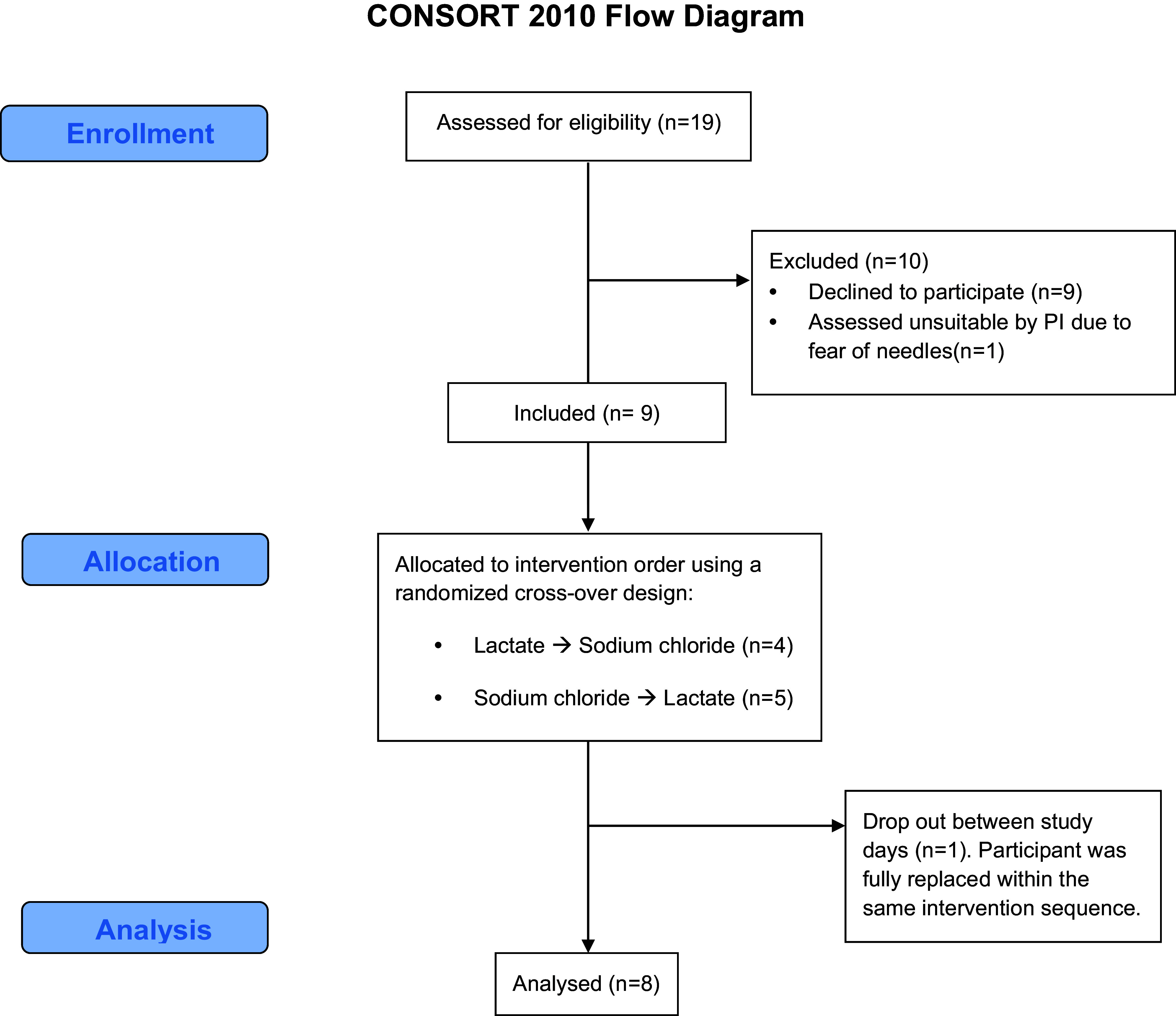
Consort flow diagram.

Eight healthy volunteers were included in the study (CONSORT flow chart, Fig. A1). Inclusion criteria were male sex (sex assigned at birth), age >18 yr, and a body mass index (BMI) between 18 and 30 kg/m^2^. Exclusion criteria were chronic or acute illness, daily use of medication, or abnormalities in the screening blood samples (sodium, potassium, creatinine, C-reactive protein, alanine transaminase, bilirubin, basic phosphatase, hemoglobin, HbA1c, and C-peptide). Each participant was studied twice, separated by at least 14 days. Enrollment took place from January to April 2021. The study was conducted from March to June 2021. There was no additional follow-up.

### Design

The study was designed as a single-blind, randomized, crossover trial. Volunteers were blinded for intervention. Randomization was done using an online randomizer (www.randomizer.org). The PI enrolled participants and assigned allocation sequences.

### Interventions

Interventions consisted of: *1*) intravenous infusion of racemic Na-Lactate (45 g lactate/L corresponding to 0.5 mol/L, Monico S.P.A, Italy) (LAC). A priming dose of 50 μmol/kg total body weight (TBW)/min was infused during the first 30 min (0–30 min), after which a continuous infusion of 25 µmol/kg TBW/min was infused during the remaining 330 min (30–360 min) of the study. *2*) Intravenous infusion of a sodium-matched (15.2 g Na^+^/L) sodium chloride solution (CTR) was given at similar infusion rates as during LAC.

### Study Days

Volunteers were instructed to abstain from strenuous physical activity, exercise, and alcohol and to eat a regular diet (∼15% fat, 30% protein, and 55% carbohydrate) for 48 h before each study day. Volunteers were studied after an overnight (10 h) fast at the Medical/Steno Aarhus research laboratory, Aarhus N, Denmark. On the study day, volunteers were instructed to arrive by bus/car/taxi to minimize physical activity. After arrival at the research laboratory, volunteers were placed in a bed, and a venous catheter was placed in the cubital vein for intravenous infusions. Another catheter was placed in an ipsilateral hand vein and kept warm using a heating pad for arterialized blood sampling (14). For deep venous blood sampling, a third venous catheter was placed retrogradely in a cubital vein on the contralateral arm (15).

**Figure 1.**
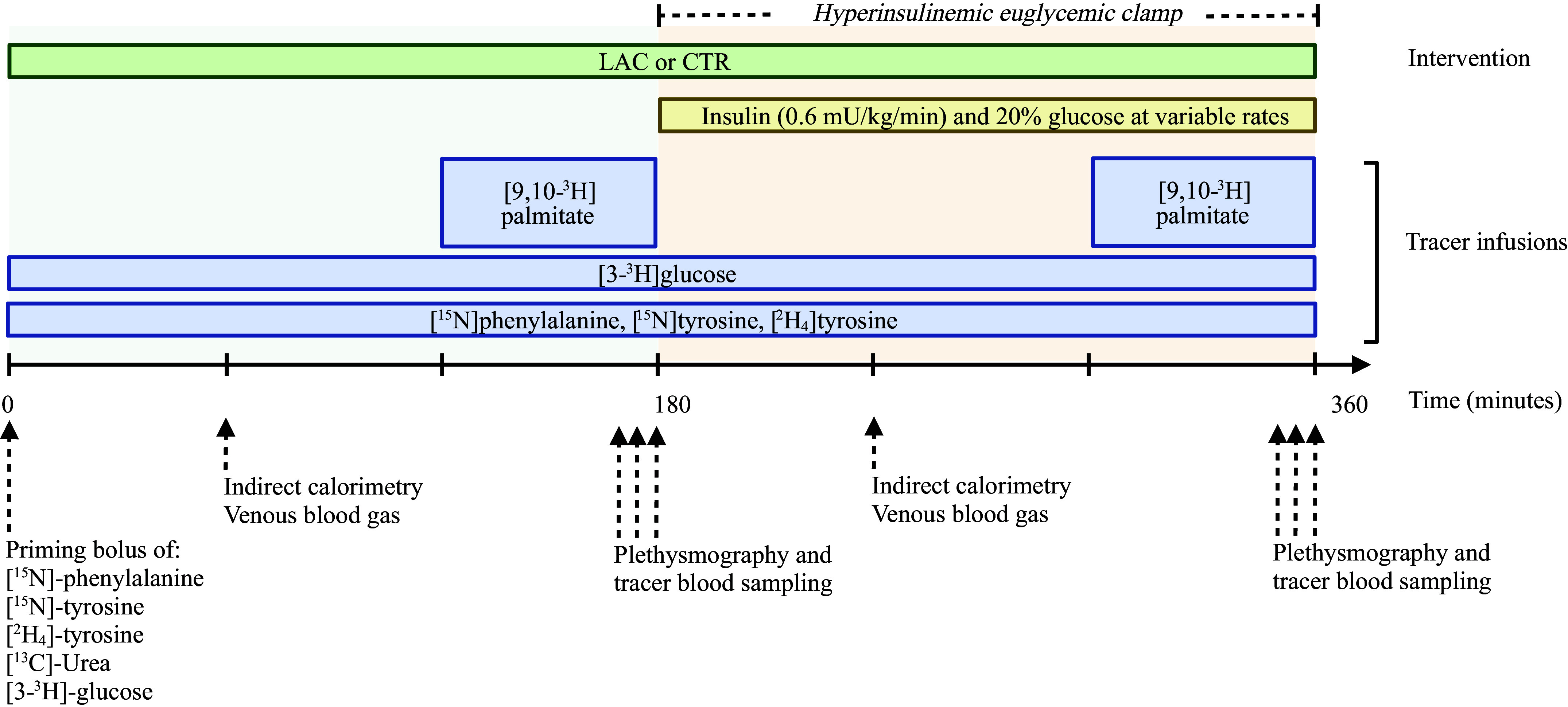
Study day flowchart. CTR, control (sodium-matched NaCl infusion); LAC, lactate (sodium-lactate infusion). Created with BioRender.com

Study days were identical apart from the interventions. Figure 1 represents the study day flow chart. Each study day consisted of a 180-min postabsorptive period (0–180 min) followed by a 180-min hyperinsulinemic-euglycemic clamp (HEC, 180–360 min).

### Hyperinsulinemic Euglycemic Clamp

A hyperinsulinemic-euglycemic clamp (HEC) from time 180 to 360 min was utilized to assess insulin sensitivity. A continuous infusion of insulin 0.6 mU/kg TBW/min (Actrapid, Novo Nordisk, Denmark) was administered during the 3-h HEC. Plasma glucose levels were clamped at ∼5 mmol/L by continuous infusion of 20% glucose at variable infusion rates based on plasma glucose concentrations measured every 10 min using a YSI 2300 model Stat Plus glucose and lactate analyzer (YSI Incorporated, Yellow Springs, OH). The *M* value was calculated based on the mean glucose infusion rate (GIR) during steady state (340–360 min) as previously described (16).

### Substrate Metabolism

At *time 0* min, primed continuous infusions of [^15^N]phenylalanine (10 mg/mL) (prime 0.75 mg/kg, continuous infusion 0.75 mg/kg/h), [^2^H_4_]tyrosine (0.6 mg/mL) (prime 0.6 mg/kg, continuous infusion 0.6 mg/kg/h), [^15^N]tyrosine (0.5 mg/mL) (bolus of 0.3 mg/kg), [^13^C]urea (10 mg/mL) (prime 390.6 mg, continuous infusion 42 mg/h), and [3-^3^H]glucose (prime 0.444 MBq, continuous infusion 0.2664 MBq/h) were initiated. During the HEC, 100 μCi [3-^3^H]glucose was added to the 500 mL of 20% glucose. [9,10-^3^H]palmitate tracer was infused between 120–180 min and 300–360 min at a rate of 0.666 MBq/h. Arteriovenous blood samples for measuring isotopic enrichments were drawn in triplicates at times *160*-, *170*-, and *180* min and again at times *340*-, *350*-, and *360* min. Blood drawings were preceded by blood flow measurements across the forearm utilizing venous occlusion plethysmography (17). Palmitate-, glucose-, and amino acid tracer kinetics were calculated as previously described (16, 18). Δ Glucose rate of disappearance (ΔRd_glu_) was calculated as the Rd_glu_ at the end of the HEC minus Rd_glu_ at the end of the postabsorptive period.

### Indirect Calorimetry

Indirect calorimetry (Oxycon Pro, Intramedic) was used to collect respiratory gases (V̇o_2_ and V̇co_2_) for ∼10 min at 60 min (postabsorptive) and 240 min (HEC). Measures of V̇co_2_ were corrected for CO_2_ retention as described in detail by others (19). Circulating HCO_3_^−^ was measured in peripheral venous blood gases (ABL800 FLEX, Radiometer). The respiratory exchange rate (RER) was calculated as V̇co_2_ (L/min) divided by V̇o_2_ (L/min). Resting energy expenditure (EE) (kcal/24 h) and oxidation rates (mg/kg TBW/min) were calculated as described by others (19). Urea excretion rates were measured in urine collected following indirect calorimetry to calculate protein oxidation rates.

### Blood Samples

The arterialized plasma l-lactate concentration was measured on-site using the YSI 2300 model Stat Plus glucose and lactate analyzer (YSI Incorporated, Yellow Springs, OH) once every hour in the postabsorptive period and with 10-min intervals during the HEC. Insulin, c-peptide, and free fatty acids were sampled hourly throughout the study day. In addition, free fatty acids were sampled in triplicates by the end of the postabsorptive period and the end of the HEC. Glucagon and cortisol were sampled hourly during the postabsorptive period and once by the end of the HEC. All blood samples were immediately placed on ice, centrifuged, and stored for later batch analyses at −20°C. Commercially available kits were used for analyses of plasma/serum concentrations of insulin (Mercodia Insulin ELISA, Sweden), C-peptide (Mercodia C-peptide ELISA, Sweden), free fatty acid (FUJIFILM Wako Chemicals Europe GmbH, Germany), glucagon (Mercodia Glucagon ELIZA, Sweden), and acyl ghrelin [Bertin Bioreagent Acylated Ghrelin (human) Express ELISA, France] in accordance with manufacturers guidelines.

### Statistics

The primary end point was a change in lipolysis rate between the two interventions measured as [9,10-^3^H]palmitate flux at 180- and 360 min. A prestudy power calculation was performed using α = 0.05, β = 0.8, and an expected reduction in lipolysis of δ = 15% and an assumed standard deviation (SD) of σ = 0.17 (20). This resulted in *n* = 8.

All statistical analyses were performed in Stata 14 (StataCorp LP, College Station, TX). Data in the text are presented as means ±SD, median (range), or mean difference (95% confidence interval, CI). Graph data are presented as means ± SD or boxplots with individual data points. Data were analyzed using a mixed model for repeated measures, followed by post hoc pairwise comparisons to compare outcomes measured at the end of the postabsorptive period (180 min) and the end of the HEC (360 min). Time, intervention, and the interaction between the two were included as fixed effects and participants as random effects. Model *P* values are included in graphs and tables: “Interaction, *P*” indicates the *P* value for the interaction between time and intervention, “intervention, *P*” indicates the *P* value for the effect of the intervention, and “time, *P*” indicates the *P* value for the effect of time. *P* values in the text represent those of the post hoc pairwise comparisons at the given time points (end of postabsorptive period or end of HEC). Model validation was conducted by inspecting scatter- and QQ-plots of the predicted versus fitted residuals. Because the *M* value and ΔRd_glu_ were measured once on each study day, they were compared between study days with a paired *t* test. *P* values <0.05 were considered significant.

## RESULTS

### Participants

Eight male participants completed the study. Baseline characteristics are presented in Table 1.

**Table 1.** Baseline characteristics

Baseline Characteristics	
Age, yr, median (range)	26 (23–29)
Weight, kg, median (range)	79 (63–92)
Height, cm, median (range)	183 (175–193)
BMI, kg/m^2^, median (range)	23.0 (20.6–26.7)
Gender	Males
Ethnicity	White/Caucasian

BMI, body mass index.

### Lactate

**Figure 2.**
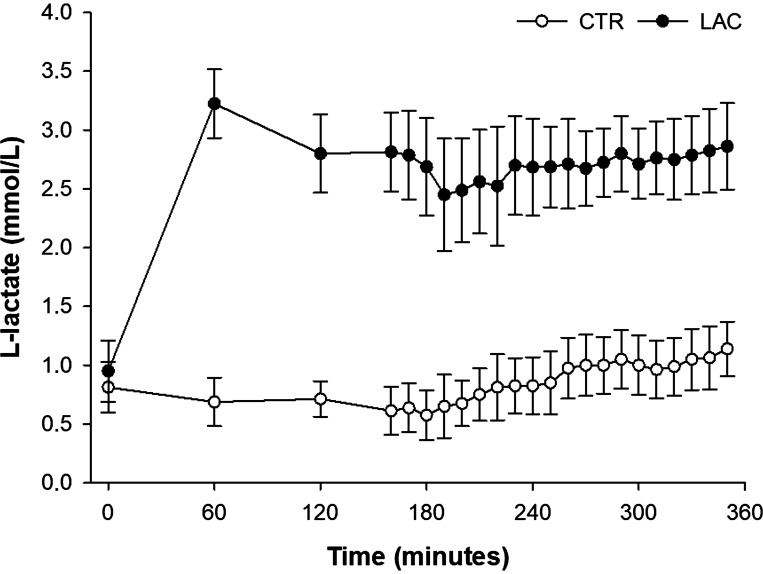
Lactate concentration. Mean plasma l-lactate concentrations ±SD (*n* = 8). CTR, control (sodium-matched NaCl infusion); LAC, lactate (sodium-lactate infusion).

The Na-lactate infusion increased plasma l-lactate to a mean of 2.7 ± 0.5 mmol/L in the postabsorptive period and 2.9 ± 0.4 mmol/L during insulin stimulation. During CTR infusion, the mean plasma l-lactate concentration was 0.6 ± 0.3 mmol/L in the postabsorptive period and 1.1 ± 0.3 mmol/L during insulin stimulation (Fig. 2).

### Lipid Metabolism

**Figure 3.**
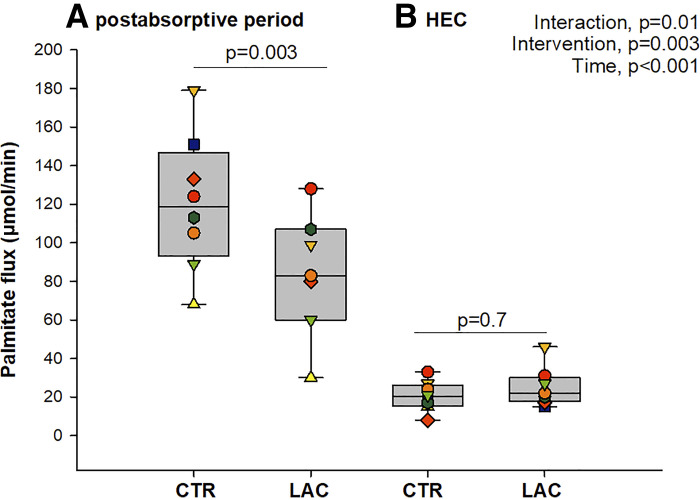
Palmitate flux. Palmitate flux (μmol/min) in the postabsorptive state (*A*) (*n* = 7, due to technical issues with the palmitate infusion in one volunteer) and during the hyperinsulinemic-euglycemic clamp (HEC, *B*) (*n* = 8). CTR, control (sodium-matched NaCl infusion); LAC, lactate (sodium-lactate infusion).

In the postabsorptive period, lipolysis rate (palmitate flux) decreased by ∼30% during LAC compared with CTR [84 ± 32 µmol/min vs. 120 ± 35 µmol/min, mean difference: −36 (−58; −14) µmol/min, *P* = 0.003, Fig. 3]. With insulin stimulation, the lipolysis rate was similarly suppressed during both LAC and CTR [25 ± 10 µmol/min vs. 21 ± 8 µmol/min, mean difference: 4 (−16; 25) µmol/min, *P* = 0.7]. At the end of the postabsorptive period, FFA concentrations were lower during LAC than CTR [mean difference: −0.1 (−0.2; −0.02), *P* = 0.02] [Table 2; Insulin administration suppressed FFA concentrations similarly during both infusions (mean difference: 0.01 (−0.1; 0.1), *P* = 0.7].

**Table 2.** Hormone- and substrate concentrations, urinary nitrogen excretion, and forearm blood flow

Hormones and Substrates	Unit	Study Period	CTR, Means ± SD	LAC, Means ± SD	Mean Difference (95% CI)	*P Value	Model
l-Lactate	mmol/L	Postabsorptive	0.6 ± 0.3	2.7 ± 0.5	2.1 (1.9; 2.3)	*P* < 0.001	Interaction: *P* < 0.001 Intervention: *P* < 0.001 Time: *P* < 0.001
		HEC	1.1 ± 0.3	2.9 ± 0.4	1.7 (1.5; 2.0)	*P* < 0.001	
Glucose	mmol/L	Postabsorptive	4.7 ± 0.3	4.8 ± 0.3	0.1 (−0.2; 0.4)	*P* = 0.5	Interaction: *P* = 0.8 Intervention: *P* = 0.8 Time: *P* < 0.001
		HEC	4.9 ± 0.3	5.1 ± 0.3	0.2 (−0.05; 0.5)	*P* = 0.2	
Glucagon	pmol/L	Postabsorptive	6 ± 2	8 ± 3	1.0 (−0.4; 2.5)	*P* = 0.2	Interaction: *P* < 0.001 Intervention: *P* = 0.5 Time: *P* < 0.001
		HEC	3 ± 1	3 ± 1	0.01 (−1.5; 1.5)	*P* = 1.0	
Insulin	pmol/L	Postabsorptive	14 ± 6	16 ± 8	2 (−20; 24)	*P* = 0.8	Interaction: *P* = 0.7 Intervention: *P* = 0.7 Time: *P* < 0.001
		HEC	174 ± 55	192 ± 16	19 (−3; 40)	*P* = 0.1	
C-peptide	pmol/L	Postabsorptive	246 ± 58	313 ± 6	67 (−6; 139)	*P* = 0.07	Interaction: *P* = 0.9 Intervention: *P* = 0.3 Time: *P* = 0.4
		HEC	270 ± 110	307 ± 139	37 (36; 110)	*P* = 0.3	
Cortisol	ng/mL	Postabsorptive	98 ± 29	79 ± 22	−19 (−39; 1)	*P* = 0.06	Interaction: *P* = 0.4 Intervention: *P* = 0.3 Time: *P* < 0.001
		HEC	80 ± 17	81 ± 16	1 (−10; 21)	*P* = 0.9	
Free fatty acids	mmol/L	Postabsorptive	0.3 ± 0.1	0.2 ± 0.1	−0.1 (−0.2; −0.02)	*P* = 0.02	Interaction: *P* = 0.03 Intervention: *P* = 0.5 Time: *P* < 0.001
		HEC	0.02 ± 0.02	0.04 ± 0.05	0.01 (−0.1; 0.1)	*P* = 0.7	
Urine urea excretion	mmol/L	postabsorptive	153 ± 81	148 ± 72	−4 (−52; 44)	*P* = 0.9	Interaction: *P* = 0.7 Intervention: *P* = 0.9 Time: *P* = 0.03
		HEC	100 ± 33	84 ± 38	−16 (−62; 31)	*P* = 0.5	
Urine nitrogen	mg/min	Postabsorptive	11 ± 5	13 ± 5	2 (−2; 6)	*P* = 0.3	Interaction: *P* = 0.3 Intervention: *P* = 0.3 Time: *P* = 0.03
		HEC	15 ± 5	15 ± 6	−1 (−5; 3)	*P* = 0.7	
Forearm blood flow	mL/100 mL/min	Postabsorptive	1.3 ± 0.7	1.1 ± 0.6	−0.3 (−0.8; 0.3)	*P* = 0.4	Interaction: *P* = 0.6 Intervention: *P* = 0.4 Time: *P* = 0.1
		HEC	0.9 ± 0.5	0.8 ± 0.4	−0.1 (−0.6; 0.5)	*P* = 0.8	

Means ± SD values and their mean difference (95% confidence interval, CI) and corresponding *P* value from post hoc pairwise comparisons at the end of the postabsorptive period (*time 180 min*) and the end of the hyperinsulinemic-euglycemic clamp (HEC) period (*time 360 min*) (*n* = 8). *P* values are based on the repeated-measures mixed model with time, intervention, and interaction between the two as fixed effects and participant as random effect. “Interaction, *P*” indicates the *P* value for the interaction between time and intervention, “intervention, *P*” indicates the *P* value for the effect of the intervention, and “time, *P*” indicates the *P* value for the effect of time. CTR, control (sodium-matched NaCl infusion); LAC, lactate (sodium-lactate infusion).

* Pairwise comparison, CTR><LAC.

### Glucose Metabolism

**Figure 4.**
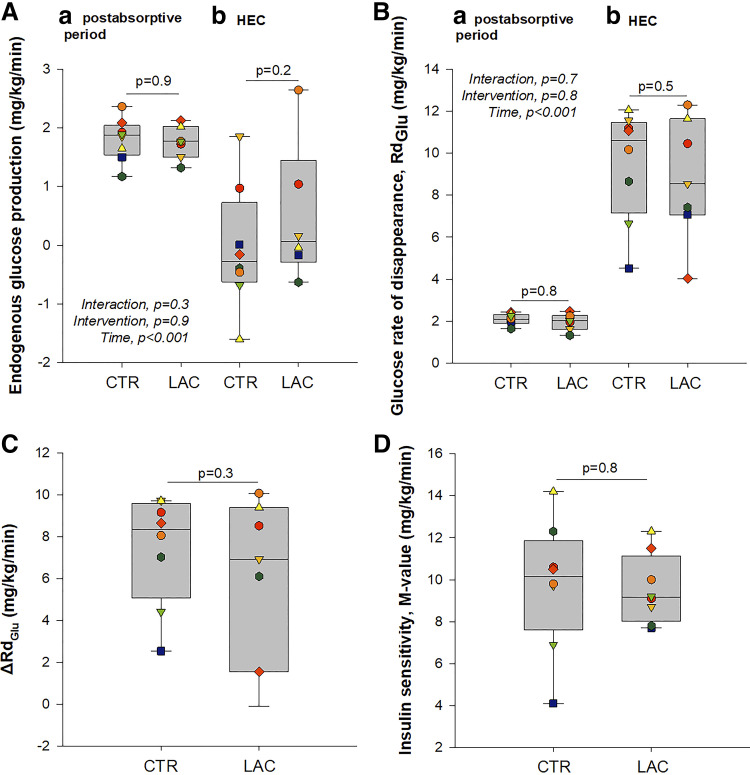
Glucose kinetics and insulin sensitivity. *A*: endogenous glucose production by the end of the postabsorptive period and the hyperinsulinemic-euglycemic clamp (HEC). Postabsorptive, *n* = 7; HEC, *n* = 6. *B*: glucose rate of disappearance (Rd_glu_) by the end of the postabsorptive period and the hyperinsulinemic-euglycemic clamp (HEC). Postabsorptive, *n* = 7, HEC, *n* = 7. *C*: ΔRd_glu_ between the postabsorptive period and the HEC, *n* = 6. *D*: insulin sensitivity (*M* value), *n* = 8. All missing data were due to technical issues with the [3-^3^H]glucose infusion. In *A* and *B*, *P* values are based on the mixed model with time, intervention, and interaction between the two as fixed effects and participants as random effect. “Interaction, *P*” indicates the *P* value for the interaction between time and intervention, “intervention, *P*” indicates the *P* value for the effect of the intervention, “time, *P*” indicates the *P* value for the effect of time. *P* values between boxplots within *a* and *b* represent post hoc pairwise comparisons based on the model. In *C* and *D*, *P* values represent paired *t* tests as there was only one ΔRd_glu_ and *M* value from each study day. CTR, control (sodium-matched NaCl infusion); LAC, lactate (sodium-lactate infusion).

There was no difference in endogenous glucose production (EGP) between LAC and CTR in the postabsorptive period [mean difference: −0.1 (−0.9; 0.8) mg/kg/min, *P* = 0.9, Fig. 4*A*]. Hyperinsulinemia had a comparable suppressive effect on EGP during LAC and CTR [mean difference: 0.6 (−0.3; 1.4) mg/kg/min, *P* = 0.2, Fig. 4*A*]. ΔRd_glu_ was similar between interventions [mean difference: 1.6 (−1.6; 4.9) mg/kg/min, *P* = 0.3, Fig. 4*C*]. Moreover, the *M* value was unaffected by LAC compared with CTR [mean difference: 0.9 (−1.9; 2.4), *P* = 0.8, Fig. 4*D*].

### Whole Body- and Forearm Muscle Phenylalanine Metabolism

**Figure 5.**
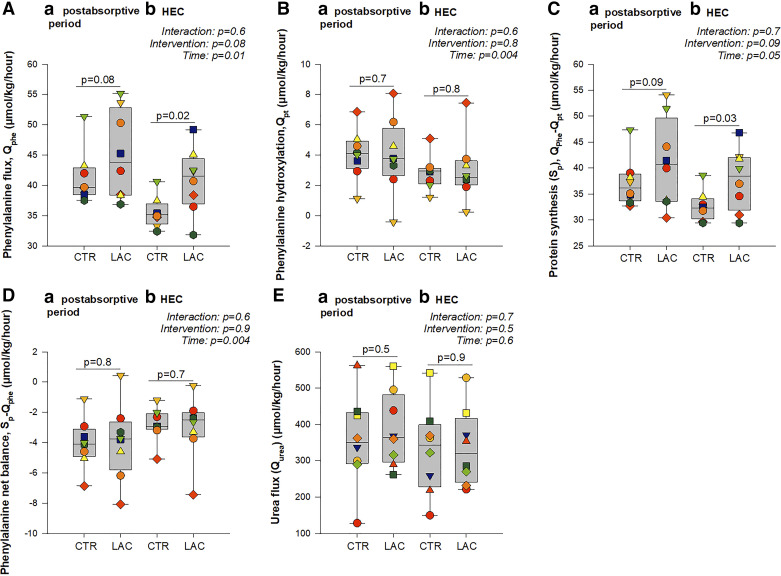
Whole body amino acid and urea kinetics. Phenylalanine flux (*A*), phenylalanine hydroxylation to tyrosine (*B*), protein synthesis (*C*), phenylalanine net balance (*D*), urea flux (*E*). *P* values are based on the mixed model with time, intervention, and interaction between the two as fixed effects and participants as random effect. “Interaction, *P*” indicates the *P* value for the interaction between time and intervention, “intervention, *P*” indicates the *P* value for the effect of the intervention, and “time, *P*” indicates the *P* value for the effect of time. *P* values between boxplots within *a* and *b* represent post hoc pairwise comparisons based on the model. *n* = 8. CTR, control (sodium-matched NaCl infusion); HEC, hyperinsulinemic-euglycemic clamp; LAC, lactate (sodium-lactate infusion); *Q*_phe_, phenylalanine flux; *Q*_pt_, phenylalanine to tyrosine hydroxylation; *Q*_urea_, urea flux; S_p_, protein synthesis.

**Figure 6.**
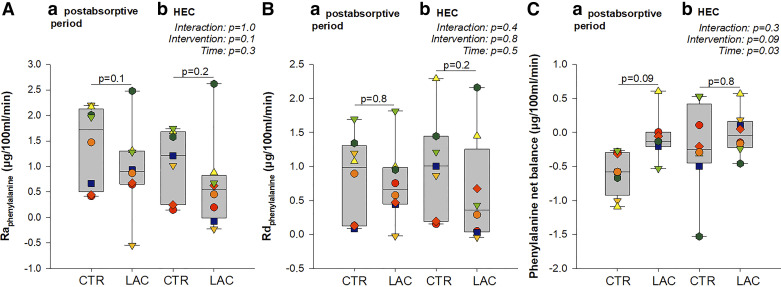
Forearm phenylalanine kinetics. Phenylalanine rate of appearance (*A*), phenylalanine rate of disappearance (*B*), phenylalanine net balance (*C*). *P* values are based on the repeated-measures mixed model with time, intervention, and interaction between the two as fixed effects and participants as random effect. “Interaction, *P*” indicates the *P* value for the interaction between time and intervention, “intervention, *P*” indicates the *P* value for the effect of the intervention, and “time, *P*” indicates the *P* value for the effect of time. *P* values between boxplots within *a* and *b* represent post hoc pairwise comparisons based on the model. All measures in the postabsorptive period, *n* = 8; measures during hyperinsulinemic-euglycemic clamp (HEC) with control (CTR, sodium-matched NaCl infusion), *n* = 7, because of saline sample dilution at the time of sample drawing for *n* = 1. LAC, lactate (sodium-lactate infusion); Ra, rate of appearance; Rd, rate of disappearance.

In the postabsorptive period, there was no significant difference in whole body phenylalanine flux between LAC and CTR (*P* = 0.09, Fig. 5*A*). However, during insulin stimulation, whole body phenylalanine flux was ∼15% higher during LAC compared with CTR (*P* = 0.02, Fig. 5*A*). Similarly, there was no significant difference in whole body protein synthesis between LAC and CTR in the postabsorptive period (*P* = 0.09, Fig. 5*C*). Still, during insulin stimulation, LAC induced ∼16% higher whole body protein synthesis than CTR (*P* = 0.03, Fig. 5*C*). Consequently, there was no difference in whole body phenylalanine net balance (Fig. 5*D*) and phenylalanine to tyrosine hydroxylation (Fig. 5*B*) between LAC and CTR in the postabsorptive period or during insulin stimulation. In addition, there was no difference in whole body urea flux (Fig. 5*E*, postabsorptive period: *P* = 0.5, HEC: *P* = 0.9) and urinary urea excretion (Table 2). Furthermore, there was no difference in forearm muscle protein net balance, protein synthesis (Rd_phe_) (Fig. 6*B*), or -breakdown (Ra_phe_) (Fig. 6*A*) between LAC and CTR in the postabsorptive period or during insulin stimulation.

### Acid-Base Status

**Figure 7.**
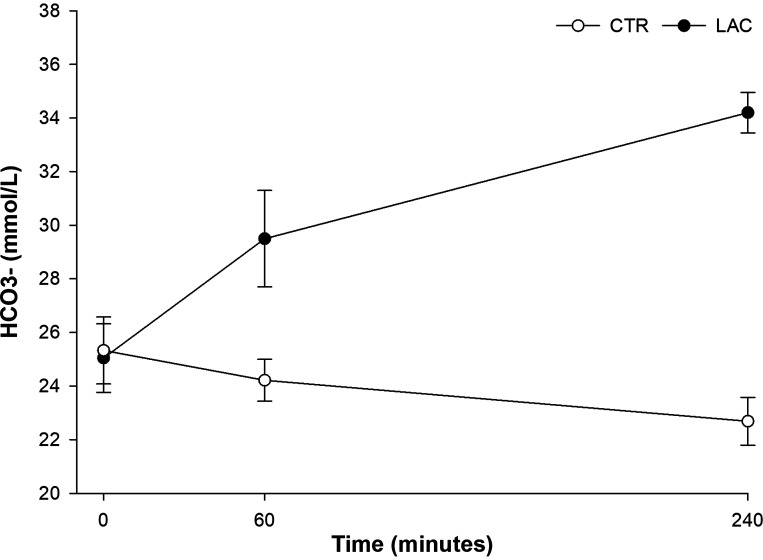
HCO_3_^−^ concentration. Mean HCO_3_^−^ concentrations ± SD [*n* = 7 for lactate (LAC, sodium-lactate infusion) and *n* = 8 for control (CTR, sodium-matched NaCl infusion)].

The HCO_3_^−^ concentrations increased during LAC (Fig. 7) from a baseline concentration of 25 ± 1 mmol/L to 34 ± 1 mmol/L after 240 min [mean difference: 9 (9; 10) mmol/L, *P* < 0.001]. In contrast, HCO_3_^−^ concentrations decreased during CTR, from 25 ± 1 mmol/L at baseline to 23 ± 1 mmol/L after 240 min [mean difference −3 (−3; −2) mmol/L, *P* < 0.001]. In addition, there was a significant increase in pH during LAC, from 7.35 ± 0.01 at baseline to 7.47 ± 0.02 after 240 min [mean difference, 0.12 (0.09; 0.15), *P* < 0.001]. Conversely, pH decreased during CTR, from 7.38 ± 0.02 at baseline to 7.34 ± 0.03 after 240 min [mean difference: −0.04 (−0.08; 0.01), *P* = 0.01].

### Indirect Calorimetry

In the postabsorptive period, RER was significantly higher on LAC than on CTR (*P* = 0.03) (Table 3). However, with hyperinsulinemia, there was no significant difference between the two study days (*P* = 1.0) (Table 3). LAC increased resting EE by ∼5% in the postabsorptive period (*P* = 0.02) and ∼3% during insulin stimulation (*P* = 0.03) compared with CTR (Table 3). Substrate oxidation rates were unaffected by interventions during both study periods (Table 3).

**Table 3.** Indirect calorimetry

Calorimetry Measure	Unit	Study Period	CTR, Means ± SD	LAC, Means ± SD	Mean Difference (95% CI)	*P* Value	Model
Resting energy expenditure	kcal/24-h	Postabsorptive	1,888 ± 183	1,984 ± 218	99 (20; 178)	*P* = 0.02	Interaction: *P* = 0.8 Intervention: *P* = 0.02 Time: *P* = 0.02
		HEC	19,87 ± 171	2,052 ± 203	87 (7; 166)	*P* = 0.03	
Respiratory exchange ratio		Postabsorptive	0.83 ± 0.1	0.93 ± 0.1	0.1 (0.01; 0.2)	*P* = 0.03	Interaction: *P* = 0.1 Intervention: *P* = 0.03 Time: *P* = 0.03
		HEC	0.92 ± 0.1	0.93 ± 0.1	−0.0 (−0.1; 0.1)	*P* = 1.0	
Carbohydrate oxidation	mg/kg/min	Postabsorptive	1.9 ± 1.6	3.3 ± 2.3	1.5 (−0.2; 3.1)	*P* = 0.08	Interaction: *P* = 0.3 Intervention: *P* = 0.08 Time: *P* = 0.09
		HEC	3.1 ± 1.6	3.5 ± 2.1	0.2 (−1.5; 1.9)	*P* = 0.8	
Lipid oxidation	mg/kg/min	Postabsorptive	0.7 ± 0.6	0.2 ± 0.8	−0.5 (−1.1; 0.1)	*P* = 0.09	Interaction: *P* = 0.2 Intervention: *P* = 0.09 Time: *P* = 0.05
		HEC	0.1 ± 0.6	0.1 ± 0.8	0.02 (−0.6; 0.6)	*P* = 1.0	
Protein oxidation	mg/kg/min	Postabsorptive	0.9 ± 0.3	1.0 ± 0.3	0.2 (−0.1; 0.5)	*P* = 0.3	Interaction: *P* = 0.4 Intervention: *P* = 0.3 Time: *P* = 0.02
		HEC	1.2 ± 0.3	1.2 ± 0.4	−0.0 (−0.3; 0.3)	*P* = 0.9	

Means ± SD values and their mean difference (95% confidence interval, CI) and corresponding *P* value from post hoc pairwise comparisons at the end of the postabsorptive period (*time 180 min*) and the end of the hyperinsulinemic-euglycemic clamp (HEC) period (*time 360 min*). *P* values are based on the repeated-measures mixed model with time, intervention, and interaction between the two as fixed effects and participant as random effect. “Interaction, *P*” indicates the *P* value for the interaction between time and intervention, “intervention, *P*” indicates the *P* value for the effect of the intervention, and “time, *P*” indicates the *P* value for the effect of time. *n* = 6, due to unavailable venous blood gases (*n* = 1 in the postabsorptive period, *n* = 2 during HEC) and missing urine collection (*n* = 1 in the postabsorptive period). CTR, control (sodium-matched NaCl infusion); LAC, lactate (sodium-lactate infusion).

### Hormones and Metabolites

Plasma glucose, insulin, C-peptide, glucagon, and cortisol concentrations were similar between LAC and CTR in the postabsorptive period and with insulin stimulation (Table 2).

## DISCUSSION

The key finding of the present study was that a lactate infusion reduced the postabsorptive rate of lipolysis and the FFA concentration. Despite this lactate-mediated decrease in released and circulating FFAs, no effects on insulin sensitivity or glucose metabolism were observed. In addition, a lactate infusion resulted in an increased rate of whole body phenylalanine (but not muscle phenylalanine) breakdown and synthesis.

The antilipolytic effects of lactate are well established in dogs (8, 9) and from in vitro studies (7, 21, 22). However, results from clinical trials have been conflicting, often tentatively inferring lactate’s effects on lipolysis from static measures of FFA- and glycerol concentrations (10–13). Only one previous study has examined the antilipolytic effects of lactate by utilizing an isotopic lipid tracer during 30 min of lactate infusion (13); this study used ^14^C-labeled oleate during 30 min in an uncontrolled design and concluded that the observed decrease in circulating FFA concentration was caused by an increased clearance, a conclusion that may have been affected by uncontrolled, nonsteady state conditions. In the current study, we used a palmitate tracer to quantify lipolysis. Our findings showed a significant decrease in lipolysis rate during the postabsorptive period when lactate was infused. During the HEC, the prevailing antilipolytic effect of insulin was evident as the rate of lipolysis was similarly suppressed on both study days. Notably, the insulin concentration did not differ between interventions, indicating that the observed decrease in palmitate flux in the postabsorptive period is not due to an insulinotropic effect of lactate. In line with this, FFA levels were lower by the end of the postabsorptive period when lactate was administered compared with sodium chloride. This occurred despite a concurrent induction of metabolic alkalosis, which per se may increase the FFA concentration (23, 24). This finding contrasts results from one previous study (11), but in line with other clinical and in vitro studies (6, 10, 13). Our results strongly support an antilipolytic effect of lactate in healthy men during postabsorptive conditions that appear to be independent of insulin. It is highly plausible that activation of the GPR81/HCA1 receptor in adipose tissue mediates this inhibition of lipolysis (1, 2, 6).

As a decrease in lipolysis is associated with enhanced insulin sensitivity (25), the lactate-induced reduction in lipolysis could theoretically improve insulin sensitivity. We used [3-^3^H]glucose and a hyperinsulinemic-euglycemic clamp to investigate insulin sensitivity and glucose metabolism. We found no effect of hyperlactatemia on EGP compared with sodium chloride in the postabsorptive period nor during the HEC. We also did not find any effect of hyperlactatemia on the increase in Rd_glu_ during hyperinsulinemia. These finding aligns with previous clinical studies (11, 26–29). Finally, we found no effect of hyperlactatemia on the *M* value. A possible explanation for why the considerable reduction in lipolysis did not result in improved insulin sensitivity could be that lactate as an alternative energy substrate may intrinsically induce insulin resistance, similar to the concept of the “glucose-fatty acid cycle” (30). Our participants were all healthy, lean, insulin-sensitive men, and whether the effect would be the same in people with insulin resistance is unknown.

We observed increased whole body phenylalanine flux (protein breakdown) and protein synthesis during the HEC with lactate infusion. Animal and in vitro studies have implicated lactate in skeletal muscle anabolism through, e.g., the mTORC1 and MAPK/ERK1/2 signaling cascades (31). We found no difference in forearm muscle phenylalanine metabolism between the LAC and CTR. This could be due to a statistical power problem and large data variability, as our data numerically suggested an increased muscle net phenylalanine balance on LAC compared with CTR (*P* = 0.09). It is also possible that the lowering of FFA concentrations with LAC may have contributed catabolically (32). Importantly, the current study’s mean systemic lactate concentration leveled off below 3 mmol/L. During intense exercise, the intramuscular lactate concentration can elevate to as much as 25 mmol/L (33). The local lactate concentration at the level of the forearm muscle is unlikely to have reached a concentration comparable with that of exercise. Nonetheless, other clinical studies have also failed to show any clear muscle anabolic effect of intravenous lactate in combination with exercise in muscle biopsies from healthy volunteers (34).

Infusion of exogenous Na-lactate causes metabolic alkalosis. The change in pH complicates the interpretation of results from indirect calorimetry because of CO_2_ trapping, and the increase in HCO_3_^−^ concentration must be acknowledged (11, 19). In previous clinical trials, exogenously induced hyperlactatemia with circulating lactate concentrations around 3 mmol/L increased energy expenditure by 6–10% (11, 26, 29, 35). In the current study, we found an increase in resting energy expenditure of 5% in the postabsorptive period and 3% during the HEC. However, measures of HCO_3_^−^ in the current study were only available from venous blood gasses, which may underestimate the HCO_3_^−^ concentration compared with arterialized blood. As such, our measures of resting energy expenditure with LAC may be too conservative and should be interpreted cautiously. Nevertheless, our finding is consistent with previous studies, suggesting an incremental effect of exogenously induced hyperlactatemia on energy expenditure in humans. Indirect calorimetry does not allow differentiation between lactate and glucose oxidation. Previous studies using lactate tracers have shown that ∼40–50% of lactate’s rate of disappearance is attributed to its oxidation (27, 36, 37). In our study, the lactate concentration approached a steady state during the 25 µmol/kg/min of lactate infusion. Assuming that 45% was oxidized, lactate oxidation contributed to ∼11 µmol/kg/min of the lactate disposal rate, leaving alternative pathways such as glycogen and lipid synthesis to account for the remaining 14 µmol/kg/min of lactate disposal.

Strengths of the present study include the crossover design in which volunteers act as their own control, thus minimizing interindividual variability. Most previous studies have used 0.9% saline as a control infusion. We chose a sodium-matched hypertonic sodium chloride solution as the control infusion to minimize the risk of tonicity confounding results. However, the lactate infusion influenced pH, which the sodium chloride infusion did not mimic. Nonetheless, alkalosis stimulates rather than inhibits lipolysis, and it has previously been shown that a sodium bicarbonate infusion increases plasma FFA and glycerol concentrations (24). As such, we do not believe that the change in pH explains our primary findings. In addition to insulin, catecholamines are important lipolysis inhibitors through α-2 adrenoreceptor activation. We did not measure catecholamines in the current study. This is a study limitation as it implies that we cannot completely dismiss that a change in catecholamine concentrations between interventions could contribute to our findings. However, in other studies, exogenous lactate treatment does not affect the adrenalin and noradrenalin concentration during a euglycemic clamp (38, 39), and lactate infusion during exercise attenuates the exercise-related release of catecholamines (40). We only included healthy, lean men, excluding us from generalizing and extrapolating our results to women and population groups characterized by a change in substrate metabolism. The current study provides novel insight into the regulation of substrate metabolism, but whether lactate has similar effects on lipolysis and protein metabolism in, e.g., people with obesity or patients with type-2 diabetes exceeded our present study aims. In conclusion, we found that exogenous lactate inhibited lipolysis by 30% but did not affect glucose metabolism or insulin sensitivity. Lactate also elevated energy expenditure and increased phenylalanine turnover during insulin stimulation.

## DATA AVAILABILITY

The corresponding author will make data available on reasonable request.

## GRANTS

This study was financially supported by the Novo Nordisk Foundation—Tandem under Grant No. NNF19OC0055002 (to N. Møller).

## DISCLOSURES

No conflicts of interest, financial or otherwise, are declared by the authors.

## AUTHOR CONTRIBUTIONS

M.G.B.P., N.R., L.C.G., E.S., and N.M. conceived and designed research; M.G.B.P., M.B., K.B.-H., and N.G. performed experiments; M.G.B.P. analyzed data; M.G.B.P., N.R., E.S., and N.M. interpreted results of experiments; M.G.B.P. prepared figures; M.G.B.P. and N.M. drafted manuscript; M.G.B.P., N.R., M.B., K.B.-H., N.G., L.C.G., E.S., and N.M. edited and revised manuscript; M.G.B.P., N.R., M.B., K.B.-H., N.G., L.C.G., E.S., and N.M. approved final version of manuscript.

## APPENDIX

The CONSORT 2010 flow diagram is shown in Fig. A1.

## References

[1] Ahmed K, Tunaru S, Tang C, Müller M, Gille A, Sassmann A, Hanson J, Offermanns S. An autocrine lactate loop mediates insulin-dependent inhibition of lipolysis through GPR81. Cell Metab 11: 311–319, 2010. doi:10.1016/j.cmet.2010.02.012. 20374963

[2] Cai TQ, Ren N, Jin L, Cheng K, Kash S, Chen R, Wright SD, Taggart AK, Waters MG. Role of GPR81 in lactate-mediated reduction of adipose lipolysis. Biochem Biophys Res Commun 377: 987–991, 2008. doi:10.1016/j.bbrc.2008.10.088. 18952058

[3] Achten J, Gleeson M, Jeukendrup AE. Determination of the exercise intensity that elicits maximal fat oxidation. Med Sci Sports Exerc 34: 92–97, 2002. doi:10.1097/00005768-200201000-00015. 11782653

[4] Martin WH 3rd, Klein S. Use of endogenous carbohydrate and fat as fuels during exercise. Proc Nutr Soc 57: 49–54, 1998. doi:10.1079/pns19980008. 9571708

[5] Achten J, Jeukendrup AE. Relation between plasma lactate concentration and fat oxidation rates over a wide range of exercise intensities. Int J Sports Med 25: 32–37, 2004. doi:10.1055/s-2003-45231. 14750010

[6] Liu C, Wu J, Zhu J, Kuei C, Yu J, Shelton J, Sutton SW, Li X, Yun SJ, Mirzadegan T, Mazur C, Kamme F, Lovenberg TW. Lactate inhibits lipolysis in fat cells through activation of an orphan G-protein-coupled receptor, GPR81. J Biol Chem 284: 2811–2822, 2009. doi:10.1074/jbc.M806409200. 19047060

[7] De Pergola G, Cignarelli M, Nardelli G, Garruti G, Corso M, Di Paolo S, Cardone F, Giorgino R. Influence of lactate on isoproterenol-induced lipolysis and beta-adrenoceptors distribution in human fat cells. Horm Metab Res 21: 210–213, 1989. doi:10.1055/s-2007-1009193. 2568975

[8] Miller HI, Issekutz B Jr, Rodahl K, Paul P. Effect of lactic acid on plasma free fatty acids in pancreatectomized dogs. Am J Physiol 207: 1226–1230, 1964. doi:10.1152/ajplegacy.1964.207.6.1226. 14251923

[9] Gold M, Miller HI, Issekutz B Jr, Spitzer JJ. Effect of exercise and lactic acid infusion on individual free fatty acids of plasma. Am J Physiol 205: 902–904, 1963. doi:10.1152/ajplegacy.1963.205.5.902. 5877419

[10] Boyd AE 3rd, Giamber SR, Mager M, Lebovitz HE. Lactate inhibition of lipolysis in exercising man. Metabolism 23: 531–542, 1974. doi:10.1016/0026-0495(74)90081-x. 4828442

[11] Ferrannini E, Natali A, Brandi LS, Bonadonna R, De Kreutzemberg SV, DelPrato S, Santoro D. Metabolic and thermogenic effects of lactate infusion in humans. Am J Physiol Endocrinol Physiol 265: E504–E512, 1993. doi:10.1152/ajpendo.1993.265.3.E504. 8214058

[12] Trudeau F, Bernier S, de Glisezinski I, Crampes F, Dulac F, Rivière D. Lack of antilipolytic effect of lactate in subcutaneous abdominal adipose tissue during exercise. J Appl Physiol (1985) 86: 1800–1804, 1999. doi:10.1152/jappl.1999.86.6.1800. 10368340

[13] Ahlborg G, Hagenfeldt L, Wahren J. Influence of lactate infusion on glucose and FFA metabolism in man. Scand J Clin Lab Invest 36: 193–201, 1976. doi:10.1080/00365517609055248. 1273497

[14] Abumrad NN, Rabin D, Diamond MP, Lacy WW. Use of a heated superficial hand vein as an alternative site for the measurement of amino acid concentrations and for the study of glucose and alanine kinetics in man. Metabolism 30: 936–940, 1981. doi:10.1016/0026-0495(81)90074-3. 7022111

[15] Coles DR, Cooper KE, Mottram RF, Occleshaw JV. The source of blood samples withdrawn from deep forearm veins via catheters passed upstream from the median cubital vein. J Physiol 142: 323–328, 1958. doi:10.1113/jphysiol.1958.sp006019. 13564439 PMC1356683

[16] Thomsen HH, Rittig N, Johannsen M, Møller AB, Jørgensen JO, Jessen N, Møller N. Effects of 3-hydroxybutyrate and free fatty acids on muscle protein kinetics and signaling during LPS-induced inflammation in humans: anticatabolic impact of ketone bodies. Am J Clin Nutr 108: 857–867, 2018. doi:10.1093/ajcn/nqy170. 30239561

[17] Cooper KE, Edholm OG, Mottram RF. The blood flow in skin and muscle of the human forearm. J Physiol 128: 258–267, 1955. doi:10.1113/jphysiol.1955.sp005304. 14392606 PMC1365856

[18] Rittig N, Bach E, Thomsen HH, Pedersen SB, Nielsen TS, Jørgensen JO, Jessen N, Møller N. Regulation of lipolysis and adipose tissue signaling during acute endotoxin-induced inflammation: a human randomized crossover trial. PLoS One 11: e0162167, 2016. doi:10.1371/journal.pone.0162167. 27627109 PMC5023116

[19] Ferrannini E. The theoretical bases of indirect calorimetry: a review. Metabolism 37: 287–301, 1988. doi:10.1016/0026-0495(88)90110-2. 3278194

[20] Guo Z, Nielsen S, Burguera B, Jensen MD. Free fatty acid turnover measured using ultralow doses of [U-13C]palmitate. J Lipid Res 38: 1888–1895, 1997. 9323598

[21] Björntorp P. The effect of lactic acid on adipose tissue metabolism in vitro. Acta Med Scand 178: 253–255, 1965. doi:10.1111/j.0954-6820.1965.tb04268.x. 5834922

[22] Dieterle P, Dieterle C, Bottermann P, Schwarz K, Henner J. The influence of lactic acid on rat adipose tissue lipolysis in vitro. Diabetologia 5: 238–242, 1969. doi:10.1007/BF01212091. 5391192

[23] Straumann E, Keller U, Küry D, Bloesch D, Thélin A, Arnaud MJ, Stauffacher W. Effect of acute acidosis and alkalosis on leucine kinetics in man. Clin Physiol 12: 39–51, 1992. doi:10.1111/j.1475-097x.1992.tb00292.x. 1541083

[24] Hood VL, Keller U, Haymond MW, Küry D. Systemic pH modifies ketone body production rates and lipolysis in humans. Am J Physiol Endocrinol Physiol 259: E327–E334, 1990. doi:10.1152/ajpendo.1990.259.3.E327. 1975988

[25] Girousse A, Tavernier G, Valle C, Moro C, Mejhert N, Dinel AL, Houssier M, Roussel B, Besse-Patin A, Combes M, Mir L, Monbrun L, Bézaire V, Prunet-Marcassus B, Waget A, Vila I, Caspar-Bauguil S, Louche K, Marques MA, Mairal A, Renoud ML, Galitzky J, Holm C, Mouisel E, Thalamas C, Viguerie N, Sulpice T, Burcelin R, Arner P, Langin D. Partial inhibition of adipose tissue lipolysis improves glucose metabolism and insulin sensitivity without alteration of fat mass. PLoS Biol 11: e1001485, 2013. doi:10.1371/journal.pbio.1001485. 23431266 PMC3576369

[26] Haesler E, Schneiter P, Temler E, Jéquier E, Tappy L. Effects of lactate infusion on hepatic gluconeogenesis and glycogenolysis. Clin Physiol 15: 581–595, 1995. doi:10.1111/j.1475-097x.1995.tb00546.x. 8590553

[27] Jenssen T, Nurjhan N, Consoli A, Gerich JE. Failure of substrate-induced gluconeogenesis to increase overall glucose appearance in normal humans. Demonstration of hepatic autoregulation without a change in plasma glucose concentration. J Clin Invest 86: 489–497, 1990. doi:10.1172/JCI114735. 2200805 PMC296751

[28] Jenssen T, Nurjhan N, Consoli A, Gerich JE. Dose-response effects of lactate infusions on gluconeogenesis from lactate in normal man. Eur J Clin Invest 23: 448–454, 1993. doi:10.1111/j.1365-2362.1993.tb00789.x. 8404995

[29] Paquot N, Schneiter P, Cayeux MC, Chiolero R, Temler E, Jequier E, Tappy L. Effects of infused sodium lactate on glucose and energy metabolism in healthy humans. Diabete Metab 21: 345–352, 1995. 8586151

[30] Randle PJ, Garland PB, Hales CN, Newsholme EA. The glucose fatty-acid cycle its role in insulin sensitivity and the metabolic disturbances of diabetes mellitus. Lancet 1: 785–789, 1963. doi:10.1016/s0140-6736(63)91500-9. 13990765

[31] Lawson D, Vann C, Schoenfeld BJ, Haun C. Beyond mechanical tension: a review of resistance exercise-induced lactate responses & muscle hypertrophy. J Funct Morphol Kinesiol 7: 81, 2022. doi:10.3390/jfmk7040081. 36278742 PMC9590033

[32] Nørrelund H, Nair KS, Nielsen S, Frystyk J, Ivarsen P, Jørgensen JO, Christiansen JS, Møller N. The decisive role of free fatty acids for protein conservation during fasting in humans with and without growth hormone. J Clin Endocrinol Metab 88: 4371–4378, 2003. doi:10.1210/jc.2003-030267. 12970312

[33] Spriet LL, Howlett RA, Heigenhauser GJ. An enzymatic approach to lactate production in human skeletal muscle during exercise. Med Sci Sports Exerc 32: 756–763, 2000. doi:10.1097/00005768-200004000-00007. 10776894

[34] Liegnell R, Apró W, Danielsson S, Ekblom B, van Hall G, Holmberg HC, Moberg M. Elevated plasma lactate levels via exogenous lactate infusion do not alter resistance exercise-induced signaling or protein synthesis in human skeletal muscle. Am J Physiol Endocrinol Physiol 319: E792–E804, 2020. doi:10.1152/ajpendo.00291.2020. 32830552

[35] Chioléro R, Mavrocordatos P, Burnier P, Cayeux MC, Schindler C, Jéquier E, Tappy L. Effects of infused sodium acetate, sodium lactate, and sodium beta-hydroxybutyrate on energy expenditure and substrate oxidation rates in lean humans. Am J Clin Nutr 58: 608–613, 1993. doi:10.1093/ajcn/58.5.608. 8237864

[36] Emhoff CA, Messonnier LA, Horning MA, Fattor JA, Carlson TJ, Brooks GA. Direct and indirect lactate oxidation in trained and untrained men. J Appl Physiol (1985) 115: 829–838, 2013. doi:10.1152/japplphysiol.00538.2013. 23788576 PMC8846964

[37] Mazzeo RS, Brooks GA, Schoeller DA, Budinger TF. Disposal of blood [1-13C]lactate in humans during rest and exercise. J Appl Physiol (1985) 60: 232–241, 1986. doi:10.1152/jappl.1986.60.1.232. 3080398

[38] Maran A, Cranston I, Lomas J, Amiel SA, Macdonald I. Protection by lactate of cerebral function during hypoglycaemia. Lancet 343: 16–20, 1994. doi:10.1016/s0140-6736(94)90876-1. 7905041

[39] Richter J, Rabe D, Duysen K, Melchert UH, Oltmanns KM. Lactate infusion increases brain energy content during euglycemia but not hypoglycemia in healthy men. NMR Biomed 32: e4167, 2019. doi:10.1002/nbm.4167. 31468650

[40] Fattor JA, Miller BF, Jacobs KA, Brooks GA. Catecholamine response is attenuated during moderate-intensity exercise in response to the “lactate clamp”. Am J Physiol Endocrinol Physiol 288: E143–E147, 2005. doi:10.1152/ajpendo.00117.2004. 15328074

